# Diagnostic Accuracy of Magnetic Resonance Spectroscopy in Predicting the Grade of Glioma Keeping Histopathology as the Gold Standard

**DOI:** 10.7759/cureus.22056

**Published:** 2022-02-09

**Authors:** Zunaira Rafique, Muhammad Wasim Awan, Shaghaf Iqbal, Naila Nasir Usmani, Mahjabeen Mahmood Kamal, Wajiha Arshad, Mashkoor Ahmad, Hassan Mumtaz, Shahzaib Ahmad, Mohammad Hasan

**Affiliations:** 1 Diagnostic Radiology, KRL Hospital, Islamabad, PAK; 2 General Medicine, Surrey Docks Health Centre, London, GBR; 3 Public Health, Health Services Academy, Islamabad, PAK; 4 Clinical Research, Maroof International Hospital, Islamabad, PAK; 5 Urology, Guy's and St Thomas' NHS Foundation Trust, London, GBR; 6 Physiology, Mayo Hospital, Lahore, PAK; 7 House Officer, Jinnah Postgraduate Medical Centre, Karachi, PAK; 8 Medical Student, Jinnah Sindh Medical University, Karachi, PAK

**Keywords:** sensitivity, specificity, magnetic resonance imaging, glioma, diagnostic accuracy

## Abstract

Background

Gliomas are the most prevalent intrinsic tumors of the central nervous system and are categorized from grade I to grade IV. Magnetic resonance imaging (MRI) provides exact diagnosis, prognosis, and assessment of tumor response to current chemotherapy/immunotherapy and radiation therapy. With histopathology serving as the gold standard, we aimed to assess the diagnostic accuracy of magnetic resonance spectroscopy (MRS) in predicting glioma grade.

Methodology

This cross-sectional study was conducted in the Department of Radiology, KRL Hospital, Islamabad, from December 15, 2019, to September 30, 2021. After providing written consent, 80 patients with untreated gliomas were included in this study. The voxel of interest was identified using MRI brain conventional contrast-enhanced sequences to assess the grade of the gliomas and link it to the histology report. Following this identification, tissue metabolites were calculated using MRS.

Results

The patients’ age ranged from 13 to 80 years, with a mean age of 49.5 years. Male patients comprised 57.5% of the total study population, while female patients comprised 42.5%. Overall, 23.75% of patients had low-grade tumors, while 76.25% had high-grade tumors. Low-grade tumors had a choline (Cho)/creatine (Cr) metabolite ratio of 1.7421, whereas high-grade tumors had an average Cho/Cr metabolite ratio of 2.5575. N-acetyl aspartate (NAA)/Cr ratio was 1.6368 in low grade and 0.6734 in high-grade tumors. Sensitivity of 77% and specificity of 84.2% were noted, with 78.75% diagnostic accuracy for the Cho/Cr ratio.

Conclusions

Multivoxel MRS has been shown to reliably predict the grade of gliomas despite its non-invasive nature and lack of procedural challenges. When used together Cho/Cr and NAA/Cr ratios and histopathology can accurately determine tumor grade and can be used as a supplementary non-invasive technique.

## Introduction

The prognosis of patients with brain tumors improves if the tumor is discovered early [1]. Gliomas are one of the most common types of brain tumors, with a five-year survival rate of less than 5%. To plan, predict, and respond to treatment, it is important to accurately predict the grade [2].

Severe problems are associated with stereotactic brain biopsies needing definitive histology in determining the grade of malignancy, including seizures, temporary or permanent neurological damage, and intracranial bleeding [3]. A sample error, which can lead to a faulty diagnosis, is also a possibility [1]. Hence, neuroimaging, such as computed tomography (CT) and magnetic resonance imaging (MRI), can be used to detect and grade brain tumors [4]. Lesion aggressiveness is indicated by MRI findings such as postcontrast enhancement, necrosis inside the tumor, and substantial perilesional edema or mass impact [1]. It is possible to use these characteristics to determine the grade of gliomas [1].

Biochemical indicators in the brain can be measured by magnetic resonance spectroscopy (MRS), which can be used in conjunction with standard MRI to predict the grade of the identified lesions. Resonance measurements of different metabolites in the brain can then be used to determine the amplitudes of these reactions [5]. MRS has high sensitivity and specificity in distinguishing malignant and benign lesions. As the grade of malignancy increases, so does the concentration of choline (Cho)-containing substances and metabolites [6]. However, because the normal brain parenchyma is replaced by tumor cells, the normal metabolites of the brain tend to diminish. Normal brain tissue produces N-acetyl aspartate (NAA), which is considered to be a metabolite [7].

Therefore, Cho concentrations and Cho/NAA ratio can be used to gauge the severity of a patient’s cancer. For this, spectroscopy can be utilized as a non-invasive alternative to biopsy to accurately estimate a tumor’s grade while avoiding the risks associated with biopsy. Using histology as the gold standard, we sought to assess the diagnostic accuracy of MRS in predicting glioma grade.

## Materials and methods

This cross-sectional validation study was carried out in the Radiology Department of KRL Hospital, Islamabad, from December 15, 2019, to September 30, 2021. In total, 80 patients with untreated gliomas were included in this study after providing written consent and undergoing surgical biopsy or resection at our hospital. All patients agreed to participate in the trial. Ethical approval was obtained from the Ethical Review Board Committee of KRL Hospital, Islamabad (reference no. KRL-HI-ERC/Dec05/19-4).

Inclusion and exclusion criteria

Space-occupying lesions on conventional MR showing malignant diffusion restriction and enhancement were included in our investigation. Patients of all ages were included in the study. However, patients under the age of 10, those who had undergone brain surgery or radiation therapy, those with known hematological/systemic conditions or stroke, and those with any other primary malignancy with suspected brain metastases were excluded from our study.

Data collection

To better understand the grade of glioma and its relationship with the patient’s histopathology report, we used MRS with tissue metabolites in conjunction with conventional MRI brain (1.5 T) contrast-enhanced sequences. This allowed us to quickly identify an important voxel for further investigation using MRS. All tumors included in the study were evaluated using the most recent World Health Organization (WHO) standards for histopathological diagnosis.

MRS with point resolved spectroscopy pulse sequence was used for data collection. The TR/TE measurement parameters utilized in scans were 2,000/46 ms with 128 signal acquisitions (Nacq). A voxel size of not less than 10 mm was chosen for the appropriate signal-to-noise ratio in the analysis. Two-dimensional magnetic resonance spectroscopic imaging (2D-MRSI) uses TR/TE of 883/43 ms, a 6 mm section thickness, and a field of view size that is tailored to the patient’s brain architecture. Transverse T2-weighted fluid-attenuated inversion recovery or T2-weighted fast spin-echo (FSE), sagittal T1-weighted FSE, and coronal T2-weighted FSE imaging sequences were used to locate a rectangular region of interest (ROI) for both spectroscopic methods.

For each patient, the peritumoral and contralateral ROIs were scanned for spectral data. The control spectrum was drawn from the contralateral normal region (cNA). As much of the solid tumor as possible was included in the voxel’s size, and placement within the tumor avoided including necrosis, cyst, hemorrhage, edema, as well as the normal-appearing brain. Baseline correction, frequency inversion, and phase shift were used after the original spectral data were post-processed. NAA, creatine (Cr), and Cho-containing compounds were the most prominent metabolites identified by proton magnetic resonance spectroscopy (^1^H-MRS).

Choline involvement in glioma biology

**Figure 1:**
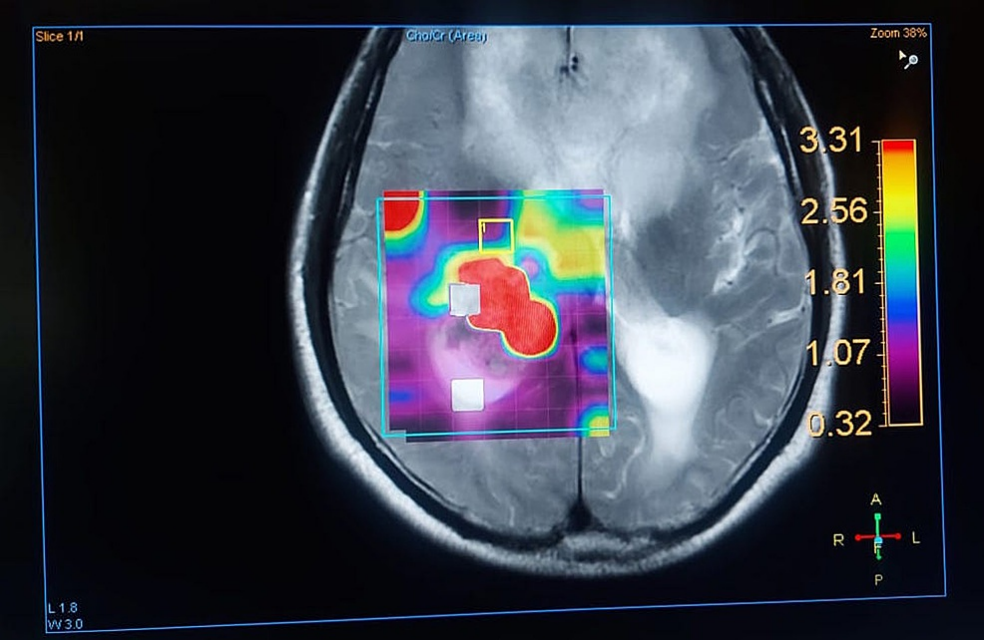
Color mapping of different metabolites according to flip angle and chemical data shift image. The choline peak at 3.3 corresponds to the red area in the map.

The concentration of Cho-containing compounds and metabolites increases as the grade of the cancer increases [6]. However, as the normal brain parenchyma is replaced by tumor cells, the brain’s natural metabolites tend to decline. NAA is a metabolite produced by normal brain tissue [7]. There is an increase in cell turnover in any high-grade tumor because Cho is an indirect measure of this turnover, which represents greater membrane synthesis and breakdown. Typical NAA myelination is seen in normal glial cells of the brain. Myelinated neurons are replaced by tumor cells when a tumor replaces the cells in question [8]. A color mapping of different metabolites according to flip angle and chemical data shift image is shown in Figure 1.

According to the WHO grading system for glioma histopathology, gliomas can be classified according to various factors, including nuclear atypia and mitosis, as well as microvascular growth and necrosis [9].

Rationale defining the two-fold change in Cho/Cr ratio

There is no two-fold change in the Cho/Cr ratio. We used the following cutoff values to grade the gliomas: for low-grade gliomas, the value was less than 2, while for high-grade gliomas, the value was greater than 2. The results were then compared to histology (grading done according to the WHO criteria) [10].

Data analysis

SPSS version 22 (IBM Corp., Armonk, NY, USA) was used to analyze the data. The metabolic ratio disparities between high and low-grade gliomas, as well as between the afflicted (intratumoral, peritumoral) and contralateral (cNA) areas, were compared using non-parametric Mann-Whitney U tests. Receiver operating characteristic curve (ROC) analysis using logistic regression models was used to determine the best cut-off values for the metabolic ratios with the highest degree of discrimination. For each parameter, sensitivity and specificity were assessed. Statistical significance was defined as P-values of 0.05 or less.

## Results

In this study, patients’ age ranged from 13 to 80 years, with a mean of 49.6 years and a median of 57 years. Overall, 57.5% of patients were male whereas 42.5% were female (Table 1).

**Table 1:** Demographic data of patients.

	Frequency (n%)
Age (years)	
13–40	9 (11.25%)
41–60	34 (42.5%)
61–80	37 (46.25%)
Gender	
Male	46 (57.5%)
Female	34 (42.5%)

Overall, 23.75% of patients suffered from low-grade tumors, whereas 76.25% developed high-grade tumors. Further classification is presented in Table 2.

**Table 2:** Classification of tumors.

Tumor	Frequency (n%)
Low grade	
Oligodendrogliomas	8 (42%)
Astrocytomas	11 (58%)
High grade	
Anaplastic astrocytomas	8 (13%)
Anaplastic oligo astrocytomas	3 (5%)
Glioblastomas	50 (82%)

**Figure 2:**
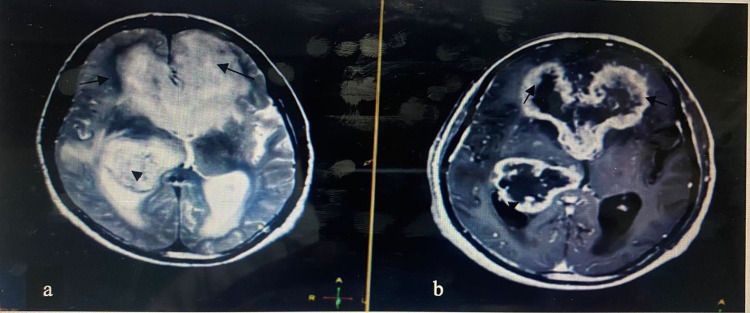
MRI brain of a 70-year-old male showing high-grade glioma (GBM) seen involving the bilateral frontal horns (arrows in a and b) and body and occipital horns (arrowheads in a and b) appearing high on T2WI and showing significant post-contrast enhancement on T2WI in b. MRI: magnetic resonance imaging; GBM: glioblastoma; T2WI: T2-weighted images

**Figure 3:**
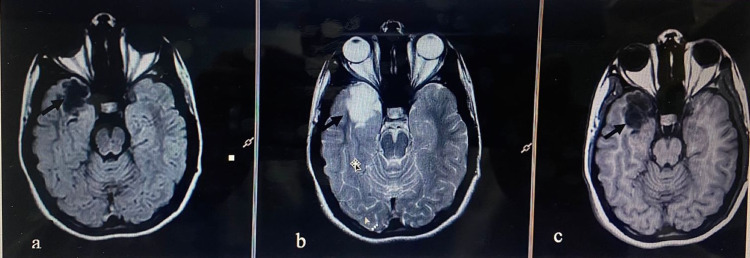
MRI with TIWI (a), T2WI (b), and FLAIR (c) showing low-grade glioma (histopathologically proven) in a 14-year-old female in the right temporal lobe (arrows in a, b, and c) appearing low on TIWI (a), high on T2WI (b), and FLAIR (c). MRI: magnetic resonance imaging; T1WI: T1-weighted images; T2WI: T2-weighted images; FLAIR: fluid-attenuated inversion recovery

Figure 2 shows a high-grade glioma (glioblastoma) with post-contrast enhancement. Figure 3 shows a low-grade glioma in the right temporal lobe using different imaging sequences.

Low-grade tumors had a Cho/Cr metabolite ratio of 1.7421, whereas high-grade tumors had an average Cho/Cr metabolite ratio of 2.5575. NAA/Cr was 1.6368 in low-grade and 0.6734 in high-grade tumors, as shown in Table 3.

**Table 3:** Average values of metabolite ratios. Cho: choline; Cr: creatine; NAA: N-acetyl aspartate

Grades of tumor	Cho/Cr	NAA/Cr
Low grade	1.7421	1.6368
High grade	2.5575	0.6734

**Figure 4:**
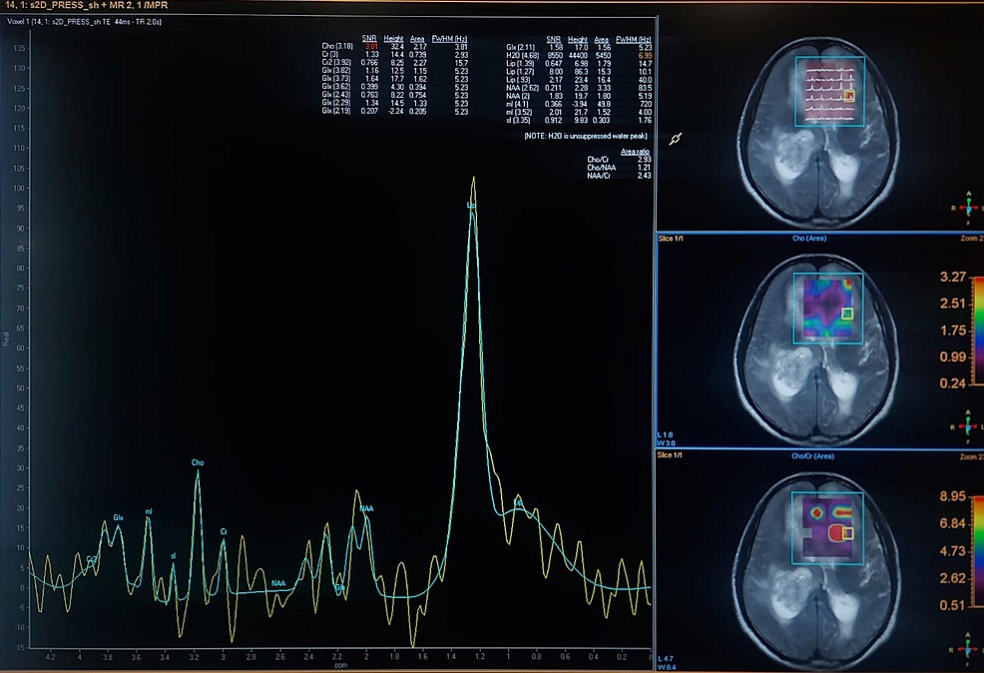
The multivoxel technique used to locate the area with a high Cho/Cr ratio of >4 (same patient as shown in Figure 2). Cho: choline; Cr: creatine

 The multivoxel technique was used to locate the area with a high Cho/Cr ratio of >4 (Figure 4).

**Figure 5:**
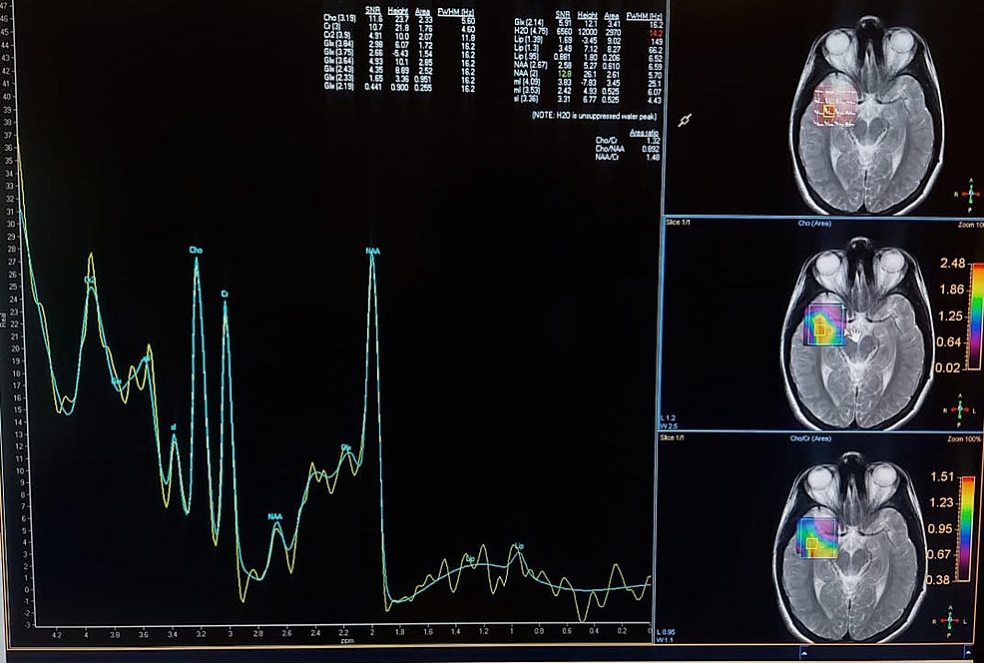
MRS using the multivoxel technique with Cho/Cr ratio of less than 2 (same patient as shown in Figure 3). Cho: choline; Cr: creatine

Figure 5 shows an MRS image using the multivoxel technique with a Cho/Cr ratio of less than 2.

Overall, 63% of patients had no lipid peak, whereas in 37% of patients a lipid peak was seen in low-grade tumors. Moreover, 57% of high-grade tumor patients had a lipid peak, leaving 43% of patients in whom no lipid peak was observed (Table 4).

**Table 4:** Differentiation of lipid peaks in low-grade and high-grade tumors.

Grades of tumor	Lipid peak		Total
	Yes	No	
Low grade	7 (37%)	12 (63%)	19 (24%)
High grade	35 (57%)	26 (43%)	61 (76%)

**Figure 6:**
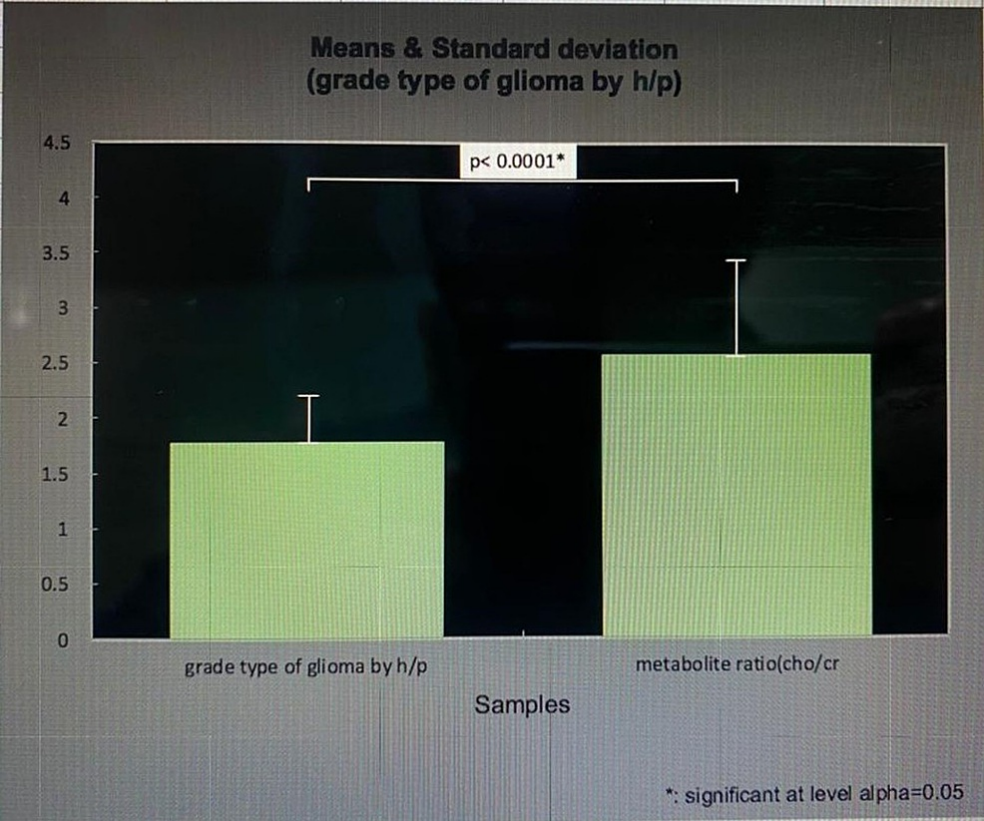
Tumor grades by histopathology in association with metabolite ratio Cho/Cr. Y-axis: Mean and standard deviation. Cho: choline; Cr: creatine

The tumor grades of glioma by histopathology had a value of 1.7625 in association with the metabolite ratio (Cho/Cr) with a value of 2.5575, and the significance of level alpha was 0.05, as shown in Figure 6.

In total, 50 patients had a Cho/Cr ratio of more than 2, of whom 47 (94%) had high-grade tumors whereas three (6%) had low-grade tumors. Thirty patients had a Cho/Cr ratio of less than 2, of whom 14 (47%) had high-grade tumors whereas 16 (53%) had low-grade tumors (Table 5).

**Table 5:** Single table analysis of MRS Cho/Cr ratio versus glioma grades by histopathology. Cho: choline; Cr: creatine; MRS: magnetic resonance spectroscopy

Metabolite Cho/Cr ratio	Histopathology grade		Total
	High grade	Low grade	
>2	47 (94%)	3 (6%)	50
<2	14 (47%)	16 (53%)	30

Our study showed 77% sensitivity and 84.2% specificity, with a 78.75% diagnostic accuracy for the Cho/Cr ratio, as shown in Table 6.

**Table 6:** Diagnostic parameters of metabolite ratio detecting both grades of gliomas. Cho: choline; Cr: creatine

Diagnostic parameters of Cho/Cr ratio detecting both grades of gliomas	Values
Sensitivity = true positive/(true positive + false negative)	77%
Specificity = true negative/(true negative + false positive)	84.2%
Positive predictive value = true positive/(true positive + false positive)	94%
Negative predictive value = true negative/(true negative + false negative)	53.3%
Diagnostic accuracy = (true positive + true negative)/all patients	78.75%

## Discussion

Since the 1970s, gliomas have been regarded as the most common type of brain tumor. Aside from cancers that are discovered by chance, better diagnostic techniques and neurosurgical facilities are considered crucial variables. The temporal lobes of the cerebral hemispheres are the most widely acknowledged location [11]. Although no single cause for gliomas has been identified, increased exposure to electric and magnetic fields, plastics, and rubber at work have been suggested as contributing factors [12].

The location of the tumor and the resulting sensory or motor loss are strongly related to the clinical presentation of these brain tumors. Patients with altered levels of awareness, chronic headaches, emesis, elevated intracranial pressure, fits, mental symptoms, or gait problems are extremely rare in this population [13]. Improved outcomes can be achieved with a timely diagnosis [14]. Contemporary diagnostic methods can be employed to detect intracranial lesions in circumstances where the patient’s condition is clinically suspected. Contrast-enhanced MRI, which uses T1, T2, and contrast-enhanced sequences, is the investigation of choice [15].

It is vital to know the grade of glioma after a diagnosis has been made as this can help doctors plan therapy and monitor the patient’s progress [16]. A stereotactic biopsy, followed by a craniotomy and an open biopsy, are the only ways to access this tissue [17].

Our study suggests an alternative to open biopsy, which could be a useful technique for predicting the grade of glioma to circumvent its consequences [17]. MRS was used to examine brain metabolites in this study. According to one study, short-time echo has a considerably higher diagnostic accuracy than intermediate-time echo. Gliomas are graded by analyzing different metabolites. Cho peak and the Cho:NAA ratio were utilized to predict the grade of gliomas in our study [18].

According to Zeng et al., Cho/NAA and Cho/Cr ratios are the best diagnostic tools for predicting the grade of gliomas [19]. An evaluation of the usefulness of MRS in determining the grade of gliomas in 2008 found that Cho and other measures related to it (Cho/Cr and Cho/NAA) were superior markers than other metabolite ratios [20].

Previous studies have reported that Cho/Cr and Cho/NAA have the largest values that can be utilized to discriminate between low-grade and high-grade malignancies taking into consideration the differences in spectroscopic approaches [21]. An American study indicated that CT and MRI contrast measurement methods are comparable in terms of assessing lesions. Due to the inability of CT and MRI to accurately diagnose canine brain tumors, histopathology remains necessary [22]. Without the need for invasive procedures, MRSI provides tumor metabolic information and insights into the physiology of malignant transformation in brain tumors. Non-invasive detection of changed isocitrate dehydrogenase gene status in gliomas, as well as cancer diagnosis and treatment response monitoring for therapeutic purposes, have been extensively researched. Radiation therapy planning can benefit from MRSI, but further research is needed to determine whether it has any real impact on clinical practice [23].

It is easy to see that the Cho:NAA ratio grows with increasing glioma grade, yet the normal parenchymal metabolite NAA declines with increasing glioma grade. As a result, MRS can be used to grade glial tumors without biopsy and the associated risks, and it does so with high sensitivity and specificity.

Limitations

The time frame and smaller sample size were the limitations of our study. Multivoxel MRS should be done on a large population for more accurate results.

## Conclusions

Multivoxel MRS can accurately predict the histological grade in patients with known gliomas because it is non-invasive and free of procedural difficulties. When determining the tumor grade, the combination of Cho/Cr and NAA/Cr is more accurate than histopathology alone.
